# Investigating the Usefulness of European Society of Cardiology Guidelines for Hospitalization in Acute Pericarditis at a Single Tertiary Center

**DOI:** 10.7759/cureus.13189

**Published:** 2021-02-07

**Authors:** João Ferreira, Mariana Luis, Rui Baptista, Sílvia Monteiro, Lino Gonçalves

**Affiliations:** 1 Cardiology, Centro Hospitalar e Universitário de Coimbra, Coimbra, PRT; 2 Rheumatology, Centro Hospitalar e Universitário de Coimbra, Coimbra, PRT

**Keywords:** hospitalization, acute pericarditis, prognosis, recurrence, guidelines

## Abstract

Background

The European Society of Cardiology (ESC) guidelines for the diagnosis and management of pericardial diseases identify predictive factors of poor prognosis and advise either in favor or against hospitalization accordingly. We aim to evaluate the adequacy of hospitalization criteria in a cohort of patients presenting to the emergency department (ED) with acute pericarditis.

Methods

Retrospective analysis of patients admitted to ED with acute pericarditis, from 2009 to 2019. During ED stay, all patients were evaluated by a cardiologist who decided if the patient was to be discharged or hospitalized. Hospitalized and discharged patients were compared regarding the primary outcome, defined by a composite of: the need for pericardiocentesis and/or cardiac surgery, pericarditis recurrence, and all-cause death. The clinical decision was then counterpoised with ESC guidelines.

Results

A total of 192 patients were included in the analysis (median age 44.5 years old, 83.3% male) of which 87 (45.5%) were hospitalized. A total of 25% registered the primary outcome, mainly due to acute pericarditis recurrence, occurring in 21.9%. Predictors of recurrence were: glucocorticoid therapy (Odds Ratio [OR]=11.93, 95% Confidence Inirtval [CI] 3.13-45.5, p<0.001), fever at admission (OR=2.67, 95% CI 1.29-5.49, p=0.008), immunosuppression (OR=4.03, 95% CI 1.280-12.659, p=0.017) and increased cardiothoracic index (OR 3.85, CI 95% 1.67-8.86, p=0.002). Regarding hospitalisation/discharge decision, the ESC guidelines were respected in 73.4% of the cases. However, no significant difference in the primary outcome was noted whether the ESC guidelines were respected or not (27.5% vs. 24.3%, p=0.707).

Conclusions

Discrepancy between current guidelines and the clinical decision did not translate into a different outcome.

## Introduction

Acute pericarditis is a common cause of non-ischemic chest pain in the emergency department (ED) [1]. In most cases, it is a benign and self-limited disease in the setting of a recent viral infection, though it can also be a life-threatening condition or leave permanent complications [2]. It may also be the case that acute pericarditis is only a manifestation of a more severe underlying condition, as a neoplasm or an immune-mediated disease [2-4].

Given this heterogeneity around acute pericarditis prognosis, the European Society of Cardiology (ESC) guidelines for the diagnosis and management of pericardial diseases identify predictive factors of poor prognosis and advise either in favor or against hospitalization of acute pericarditis patients according to their presence [5]. According to the ESC guidelines, predictors of poor prognosis can be divided into major risk factors - high fever, subacute course (symptoms over several days without a clear acute onset), evidence of large pericardial effusion (defined as the diastolic diameter of echo-free space over 20 mm), cardiac tamponade, failure of response after seven days of non-steroidal anti-inflammatory drugs (NSAIDs), and minor risk factors - myopericarditis, immunodepression, trauma and oral anticoagulant therapy [5]. The presence of one or more risk factors (major or minor) or a suspected underlying etiology (non-viral, non-idiopathic) warrants hospital admission.

However, due to local factors, the risk of nosocomial infections and the shortage of hospital beds, the ESC guidelines recommendations for admission or discharge may not be followed, also because there is a global perception of the overall good prognosis of acute pericarditis. In this study, our aim was to evaluate the adequacy of the hospitalization criteria suggested by the aforementioned ESC guidelines in a cohort of patients presenting to the ED with acute pericarditis.

## Materials and methods

We conducted a retrospective, observational cohort study including all patients admitted to ED with a clinical diagnosis of acute pericarditis, between January 2009 and May 2019. The study was conducted at a university tertiary hospital with a large ED (on average, 200,000 episodes in 2017). All patients were evaluated during the ED stay by the cardiologist on duty at the time, who ultimately decided if the patient was to be discharged or hospitalized according to his clinical observation. This observation was then confronted with the ESC guidelines recommendation for admission or discharge of the specific patient clinical setting. Patients aged 17 years or younger, diagnosed during hospitalization and evaluated by a non-cardiologist physician during ED stay, were excluded from the study.

We examined demographic and clinical characteristics at ED admission and discharge. Comorbidities were selected in categories of cardiovascular disease based on the International Statistical Classification of Diseases and Related Problems (ICD-10). Abnormal chest radiography was defined as any change visualized in the exam possibly related to acute pericarditis, such as pleural effusion, increased cardiothoracic index and signs of infection or malignancy.

We adopted the classification of pericardial effusion reported by Weitzman et al [6]: a small pericardial effusion (PE) is an echo-free pericardial space (sum of the anterior and posterior effusion) < 10 mm, moderate pericardial effusion is an echo-free space of 10 to 20 mm, and a large pericardial effusion is an echo-free space of > 20 mm. The thickness of pericardial effusion was measured and end-diastole confirmed by the onset of the QRS complex. In the case of circumferential effusions, echo-free space was measured in the parasternal long-axis view. For loculated or asymmetric effusions, the pericardium was evaluated in different planes, including off-axis views, recording the largest site and size of the effusion.

Cardiac troponin I (cTnI) was detected by a high-sensitivity assay. This assay uses cTnI-specific monoclonal antibodies directed to different epitopes being able to recognize multiple modifications in circulation [7]. A positive cTnI value was defined when the titer was ≥ 34 ng/L for men and ≥ 16 ng/L for women. Antinuclear antibodies (ANA) were measured by indirect immunofluorescence on HEp-2 (human epithelial type 2) cells in line with current guidelines [8]. ANA positivity was defined with a titer of ≥ 1:160.

**Figure 1:**
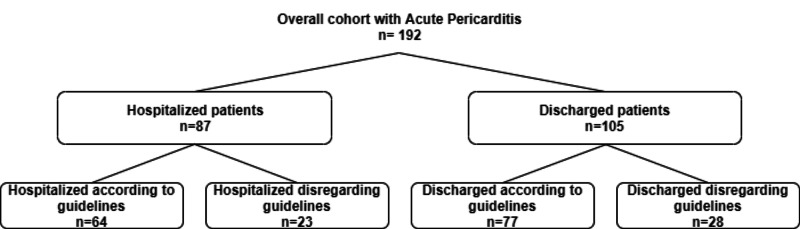
Flowchart of the study population

Patients were classified according to the: (1) clinician’s decision of hospitalization versus non-hospitalization and (2) fulfillment of ESC hospitalization criteria, as shown in Figure 1.

We defined a primary outcome consisting of the composite of (1) need for pericardiocentesis and/or cardiac decompressive surgery, (2) recurrent pericarditis, and (3) all-cause death during the follow-up period (median patient follow-up of 4.0 years ± interquartile range 5.25), and compared the aforementioned groups. Recurrent pericarditis was defined according to ESC guidelines as recurrence of pericarditis after a documented first episode of acute pericarditis and a symptom-free interval of four to six weeks or longer [5].

Quantitative data were expressed as means ± standard deviation or median with the interquartile range as appropriate and categorical data as frequencies and percentages. Groups were compared using the Student T-test or Mann-Whitney U test for continuous variables and Pearson’s χ2 test for categorical variables. Time to primary outcome distribution was estimated with the Kaplan-Meier method employing the log-rank test. To evaluate potential predictors of unfavorable outcomes, we performed multivariate analysis employing logistic regression with the forward conditional method. Data was analyzed using IBM® SPSS® Statistics, version 22.0 software (IBM Corp., Armonk, NY, USA).

The authors had full access to data and take full responsibility for its integrity.

## Results

Study population

We included 192 patients, of which 45.3% (n=87) were hospitalized (Table 1). The median age was 44.5 years (interquartile range 30.5) and 83.3% were male. Other clinical and demographic features are presented in Table 2. All 192 patients fulfilled the ESC diagnostic criteria for acute pericarditis [5], with pleuritic chest pain being the most common symptom, present in 99.0% of cases. Typical ECG changes were found in 80.5% of patients and were more common in males than females (83.7% vs. 65.6%, p=0.027). Transthoracic echocardiography was performed in 81% of patients (n=156) and PE was present in 50.6% of cases (n=79), usually mild-moderate (88.9%), and much more prevalent in females (82.1% vs. 43.8%, p<0.001). Idiopathic acute pericarditis was the most prevalent etiology (74.5%), followed by a recent history of flu-like disease or respiratory tract infection (20.3%) (Table 3).

**Table 1:** Study groups ESC: European Society of Cardiology.

		Hospitalized patients (according to clinician)		
		Yes	No	Total
Patients fulfilling hospitalization criteria (according to ESC guidelines)	Yes	64	28	92
	No	23	77	100
	Total	87	105	192

**Table 2:** Clinic-demographic features of the study population ECG: electrocardiogram; IQR: interquartile range. *79 patients of the 156 patients that underwent transthoracic echocardiography.

Feature	Total (n=192)	Hospitalized patients (n=87)	Not hospitalized patients (n=105)	p-value
Age, median (IQR), years	44.5 (30.5)	44.0 (30.0)	45 (30.0)	0.437
Male sex, n (%)	160 (83.3)	71 (81.6)	88 (84.6)	0.698
Chest pain, n (%)	189 (99.0)	85 (97.7)	104 (100.0)	0.206
Pericardial effusion, n (%)	79 (50.6)*	48 (61.5)	32 (40.0)	0.007
Large pericardial effusion, n (%)	9 (5.7)	9 (11.5)	0 (0)	0.001
Pericardial rub, n (%)	28 (14.6)	18 (20.7)	10 (9.6)	0.040
ECG changes, n (%)	148 (80.5)	65 (78.3)	83 (82.2)	0.577
Diabetes, n (%)	16 (8.6)	8 (9.8)	8 (7.8)	0.793
Arterial hypertension, n (%)	48 (26.3)	22 (26.8)	26 (25.2)	0.867
Dyslipidemia, n (%)	35 (19.4)	17 (20.7)	18 (17.5)	0.578
Known atrial fibrillation, n (%)	6 (3.2)	4 (4.9)	2 (1.9)	0.408
Known hypothyroidism, n (%)	3 (1.6)	1 (1.2)	2 (1.9)	1.000
Chronic kidney disease, n (%)	3 (1.7)	3 (4.1)	0 (0)	0.071

**Table 3:** Etiology of acute pericarditis *Without a known specific cause/assumed viral cause. ^†^Previous recent cardiac surgery or cardiac device implantation. ^Ŧ^Known, diagnosed during hospitalization or diagnosis made < 12 months after pericarditis episode.

Feature	Total patients (n=192)	Hospitalized patients (n=87)	Not hospitalized patients (n=105)	p-value
Idiopathic*, (%)	142 (74.0)	63 (72.4)	79 (76)	0.620
Hypothyroidism, n (%)	3 (1.6)	1 (1.2)	2 (1.9)	1.000
HIV infection, n (%)	2 (1.0)	0 (0)	2 (1.9)	0.501
Post-cardiac intervention^†^, n (%)	8 (4.2)	5 (5.7)	3 (2.9)	0.472
Dressler syndrome, n (%)	4 (2.0)	3 (3.4)	1 (1.0)	0.332
Chronic kidney disease, n (%)	3 (1.6)	3 (3.4)	0 (0)	0.093
Recent flu-like/ respiratory tract infection, n (%)	39 (20.3)	20 (23.0)	19 (18.3)	0.473
Neoplasia^Ŧ^, n (%)	11 (5.7)	5 (5.7)	6 (5.8)	1.000
Immunomediated disease^Ŧ^, n (%)	19 (9.9)	6 (6.9)	13 (12.5)	0.231

At ED admission, all patients were tested for a complete blood count, C-reactive protein (CRP), high-sensitivity cardiac troponin I (hs-cTnI), and ECG. Later, as part of the etiological investigation, 25.5% (n=49) underwent ANA testing, 28.6% (n=55) thyroid function tests and 19.3% (n=37) cardiac magnetic resonance (CMR) or chest computed tomography (CT) scan (Table 4). A total of 6.3% (n=12) of patients underwent emergent coronary angiography for the suspicion of acute myocardial infarction, with no culprit coronary lesions found.

**Table 4:** Analytical and imaging tests performed ANA: antinuclear antibodies; CMR: cardiac magnetic resonance; CRP: C-reactive protein; ESR: erythrocyte sedimentation rate; hs-cTnI: high-sensitivity cardiac troponin I; SD: standard deviation.

Feature	Total patients (n=192)	Hospitalized pateints (n=87)	Not hospitalized patients (n=105)	p-value
CRP, mean (SD), mg/dL	7.5 (± 9.0)	11.8 (± 10.3)	3.7 (± 5.4)	<0.001
ESR, mean (SD), mm/1^st^ hour	44.8 (± 24.5)	48 (± 25.2)	39.5 (± 23.4)	0.377
Leukocyte count, mean (SD), x10^6^/mm^3^	11.3 (± 4.6)	12.3 (± 3.7)	10.6 (± 5.1)	0.009
Positive hs-cTnI, n (%)	35 (18.2)	31 (36.5)	4 (3.8)	<0.001
Positive ANA, n (%)	25 (13.0)	12 (40.0)	13 (68.4)	0.079
Abnormal chest X-ray (%)	50 (26.0)	33 (55.0)	17 (23.9)	<0.001
Performed CT scan (%)	26 (13.5)	12 (13.8)	14 (13.5)	1.000
Performed CMR (%)	11 (5.7)	8 (9.2)	3 (2.9)	0.115

Pericardiocentesis was performed in four patients (2.1%): two patients in the context of cardiac tamponade and two other patients as a diagnostic procedure. Cardiac surgery with the creation of a pericardial window was performed in two cases due to the recurrence of a large, symptomatic pericardial effusion.

NSAIDs were the most prescribed drugs (95.7%), while combination therapy with NSAIDs and colchicine was used in 55.1% of patients. Glucocorticoids were prescribed in 7.0% of patients, of whom 61.5% had an underlying disease potentially associated with acute pericarditis.

Primary outcome

**Figure 2:**
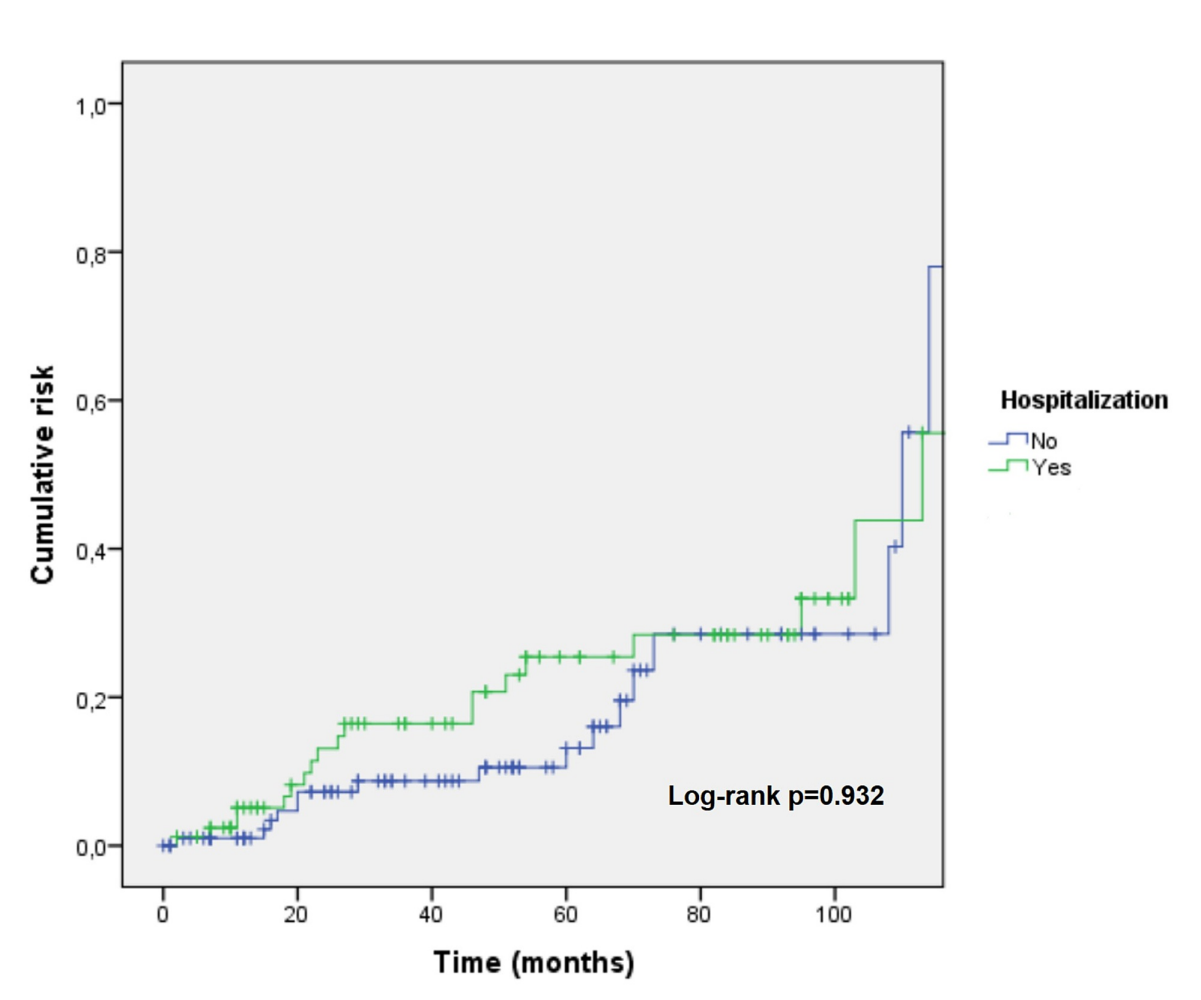
Kaplan-Meier curve depicting cumulative risk of recurrence of acute pericarditis between hospitalized and non-hospitalized patients

The median admission length was 5.0 days (interquartile range 5.0). There were no deaths during ED stay or admission. Five patients (2.6%) died during the follow-up period for reasons not related to pericarditis or its complications: two deaths related to a malignant neoplasm, one to human immunodeficiency virus (HIV) infection complications, one to severe sepsis, and one to heart failure. The primary outcome occurred in one-quarter of patients (n=48), essentially driven by pericarditis recurrence. Recurrent pericarditis comprised 87.5% of the primary outcome events. Pericardiocentesis was performed in three cases and two patients required cardiac surgery. Having at least one ESC major risk criteria was predictive of recurrence (OR 3.14, 95% CI 1.59-6.19, p=0.001). The same did not apply for minor criteria (OR 1.47, 95% CI 0.69-3.14, p=0.315). In univariate analysis, predictors of recurrence were: glucocorticoid therapy usage (OR 11.93, 95% CI 3.13-45.5, p<0.001), fever on admission (OR 2.67, 95% CI 1.29-5.49, p=0.008), drug-induced or HIV-associated immunosuppression (OR 4.03, 95% CI 1.280-12.659, p=0.017), and abnormal chest radiography (OR 5.42, CI 95% 2.33-12.62, p<0.001), particularly increased cardiothoracic index (OR 3.85, CI 95% 1.67-8.86, p=0.002). Recurrence rate and time to recurrence did not differ between hospitalized and non-hospitalized patients (log-rank p=0.932), as shown in Figure 2.

The use of colchicine became more frequent after 2015 with the publication of the most recent guidelines (79.2 % vs. 24.1 %, p<0.001). Despite not being significantly different, the change in management led to a numerical reduction in the primary outcome (16.7% vs. 24.5%, p=0.213).

ESC-guidelines admission criteria analysis

Regarding hospitalization/non-hospitalization decision, the ESC guidelines recommendations were followed in the large majority (73.3%, n=140) of the cases (Table 1). Conversely, translated into 26.7% (n=51) of patients being admitted without criteria or discharged while having at least major or minor risk factor. Almost half the patients (47.9%, n=92) fulfilled admission criteria and a similar proportion was indeed hospitalized (45.5%, n=87). However, among admitted patients, only 72.4% (n=63) fulfilled the admission criteria. On the other hand, 30.8% (n=28) patients who fulfilled admission criteria were discharged from the ED. Notwithstanding the disregard of ESC guidelines recommendations in this cohort of acute pericarditis patients, we found no significant differences in the primary outcome whether the ESC guidelines were followed or not (27.5% vs. 24.3%, p=0.707, respectively). Regarding a single component of the primary outcome, pericarditis recurrence, its incidence was also similar between the two groups (25.5% vs. 21.4%, p=0.561).

Regarding the patients discharged from the ED, there was a non-significant numerical reduction in the primary outcome in the group of patients who were correctly discharged following guidelines (14.5% vs. 28.6%, p=0.150).

Finally,, regarding patients ultimately admitted to the hospital, the primary outcome was not different when clinicians followed guidelines criteria for patient hospitalization comparing to non-recommended hospitalizations (28.6% vs. 13.6%, p=0.251).

## Discussion

In this study, we assessed the prognosis and the adequacy of ESC-guidelines defined admission criteria in a cohort of patients presenting to the ED with acute pericarditis.

We found, as described in the literature, that patients with acute pericarditis have an excellent prognosis with rare complications and almost no mortality. However, we still tend towards hospitalization of these patients, even in the total absence of predictors of poor prognosis. Acute phase markers (mainly CRP and leukocyte count), although not considered predictors of poor prognosis [5], were likely to contribute to hospitalization decision, as evidenced by higher levels among hospitalized patients.

Among non-hospitalized patients who fulfilled criteria for hospitalization, the prognosis remains excellent and similar to the ones who were hospitalized. This raises the question of whether some of these patients really need to be hospitalized instead of keeping a follow-up on an outpatient basis.

The clinic-demographic characteristics of our cohort were similar to the previous series. As previously shown, pericarditis has a higher incidence in a middle-aged population, has a male preponderance, a recurrence rate around 15-30% and a low major complication rate [9-11].

The absence of deaths directly attributed to acute pericarditis is also in line with other studies, as evidenced by a Spanish study where no deaths were registered [12], making it difficult to identify predictors of poor outcome and mortality. Nevertheless, in a Finnish study that included 1,361 admissions for acute pericarditis, increased age and severe coinfection were strong predictors of in-hospital mortality [13]. This seems to imply that, while acute pericarditis has an overall excellent prognosis, a nosocomial infection arising from patient hospitalization is associated with a worse prognosis, suggesting that hospitalization may be harmful in some cases.

Very few studies focus on the decision of hospitalization versus discharge in acute pericarditis patients, although the current ESC guidelines specifically address this issue. One Australian study, including 179 patients with clinic-demographic features similar to our cohort, mentions a 26.8% hospitalization rate, which is quite low compared to ours [14]. In this study, hospitalization decision was based on the number of risk factors present. Still, no comparison is made regarding the outcome of hospitalized versus discharged patients.

Although the ESC guidelines and the clinical reasoning regarding admission indication were consistent most of the time, in almost a quarter of the cases they differed. Still, this did not translate into a different outcome, raising the question of whether those patients really took benefit from hospitalization.

The answer to this question will depend on whether we, as clinicians, are more focused on the prevention and monitoring of acute pericarditis complications or the etiological investigation of a possible underlying and treatable disease.

The yield of hospital investigation of the etiology of acute pericarditis was low, as we established the diagnosis of idiopathic pericarditis in 74% of cases. This finding goes in line with other studies in which the underlying etiology was found in about 14 to 22% of the cases and the vast majority of cases were related to infectious etiology or other unknown causes [11]. The most common specific causes described in published clinical studies with unselected populations are neoplastic (5.1 to 7.0%), tuberculosis (3.8 to 4.0%), and autoimmune (1.7 to 7.3%). In our study, underlying etiologies are similar to the literature, except for autoimmune cause, which was slightly higher compared to other studies. This could be explained by the low median age of our sample, where autoimmune diseases are more common.

Moreover, if we add other factors as nosocomial infection risk and the limited capacity of hospital beds, we may consider a close follow-up of acute pericarditis patients in an outpatient regime as a plausible alternative to hospitalization, mainly in a population susceptible to in-hospital complications such as the immunocompromised and the elderly. Outpatient therapy of low-risk acute pericarditis cases was already tested in other centers, with good efficacy and no serious complications after a mean follow-up of 38 months, with aspirin failure being the main cause for hospitalization of those patients [15].

Recurrent pericarditis was the most common complication, affecting around 20% of the patients, which is consistent with other series described in the literature [2, 11, 16-22]. However, our follow-up period was longer (with a median follow-up of 48 months) than most studies (18 to 31 months described in the previous series) [9-11].

In general, the predictors of poor prognosis were in accordance with the ones presented in ESC guidelines [5]. In our study, the use of glucocorticoids was the strongest predictor for recurrence. This association has already been described in previous studies [2, 10, 23], especially during glucocorticoid tapering. This finding is also explained by a higher likelihood of choosing glucocorticoid therapy in patients with an underlying immune-mediated disease, which is a risk factor for recurrence and poor prognosis per se [18, 20, 24, 25].

It is important to notice that the current ESC guidelines for the diagnosis and management of pericardial diseases were published in 2015 [5], which conditioned some of our results. For instance, colchicine was only used in nearly half of the cases. This is attributed to the fact that, before 2015, the use of colchicine in acute pericarditis had a class IIa indication and level of evidence B [26]. Only after the COPE trial in 2005 [10] and ICAP trial in 2013 [9], colchicine, in association with NSAIDs, became the therapy of choice. In our cohort, there was a significant increase in the prescription of colchicine after the publication of the most recent guidelines. However, in our cohort, the paradigm shift in treatment led only to a non-significant trend towards the reduction of events. Still, we believe that the lack of significance is explained by our small sample size.

Our study has some limitations: (1) its design as a single-center, retrospective study with a relatively small number of patients is responsible for the low number of follow-up events, apart from recurrence; (2) not all patients underwent echocardiogram at admission in the ED, mainly because these patients had enough clinical data to establish a clinical diagnosis of acute pericarditis; (3) as the decision of different cardiologists may vary, ultimately criteria for hospitalization was not uniform; (4) etiology and outcomes may vary compared to other regions since tuberculosis is a quite common cause of pericarditis in developing countries; and (5) while the median follow-up period in our study was four years, the study population was comprised of patients presenting as late of May 2019, so the patients at the later end may have had significantly less follow up duration.

Our study main strength is the fact it was conducted at a tertiary hospital, which explains the vast variety of etiologies encountered. Regardless of the etiology, all patients had their diagnosis confirmed by a cardiologist. Also, as far as we are aware, this is the first study comparing the outcomes of hospitalized and discharged acute pericarditis patients. However, further prospective and multi-center studies and those which address individual major and minor risk factors are needed to support our conclusions or even update guidelines, which were based on a high level of evidence.

## Conclusions

Discrepancy between current guidelines and clinical reasoning did not translate into different outcomes. Although guidelines should always guide clinical decision-making, local factors and clinical judgment have to be taken into account when it comes to the decision to hospitalize or discharge a patient with acute pericarditis. More data is needed in order to confirm our findings or update guidelines.
